# Improved running gait parameter estimation from single foot-mounted IMU data based on refined event detection

**DOI:** 10.3389/fbioe.2025.1714473

**Published:** 2026-01-13

**Authors:** Yiwei Wu, Haoran Zhang, Shuhan Wang, Changda Lu, Qingjun Xing, Lixin Sun, Yanfei Shen

**Affiliations:** 1 School of Sport Science, Beijing Sport University, Beijing, China; 2 AI Sports Engineering Lab, School of Sports Engineering, Beijing Sport University, Beijing, China; 3 Sports Data Center of China, Beijing Sport University, Beijing, China; 4 Key Laboratory for Performance Training and Recovery of General Administration of Sport, Beijing Sport University, Beijing, China; 5 Engineering Research Center of Strength and Conditioning Training Key Core Technology Integrated System and Equipment, Ministry of Education, Beijing Sport University, Beijing, China

**Keywords:** gait event detection, inertial measurement units, running gait analysis, validation, zero-velocity update

## Abstract

**Background:**

Inertial measurement units (IMUs) enable portable gait monitoring, yet their accuracy relies on precise event detection. Conventional algorithms using raw signal peaks often fail during running due to speed variations and diverse foot-strike patterns. Therefore, adaptive detection strategies are required for high precision running gait analysis.

**Methods:**

This study proposes MFD-GED (multi-sensor fusion with dynamic gait event detection), a refined method for accurate running gait analysis via a single foot-mounted IMU. To enhance event detection, the framework fuses acceleration- and angular-velocity features and employs a parametric strategy to identify initial contact (IC), terminal contact (TC) and mid-stance (MS), respectively. The algorithm then computes a comprehensive set of gait parameters relevant to running biomechanics assessment. Data were collected from 15 healthy male runners (age: 24.1 ± 1.1 years) performing 10-m running trials. The proposed method was benchmarked against a conventional angular-velocity-based gait-segmentation algorithm (AVGS) and validated using a laboratory reference (LAB) comprising an optical motion-capture and force-plate system. Pearson correlation coefficients (Pearson’s r), intraclass correlation coefficients (ICCs), and Bland-Altman analysis were used to assess concurrent validity, while paired t-tests and Cohen’s d were employed to evaluate the performance improvement over the AVGS method.

**Results:**

The MFD-GED method demonstrated high concurrent validity against the LAB system (r = 0.743–0.991; ICC = 0.741–0.990). Compared to the AVGS method, systematic bias was reduced for spatial parameters ( p>0.05 ), including stride velocity (−0.023 m/s vs. −0.012 m/s) and stride length (0.018 m vs. 0.009 m). For temporal parameters, bias significantly decreased ( p<0.01 ; Cohen’s d = 1.62–2.20), specifically for contact time (0.057 s vs. 0.001 s) and flight time (−0.063 s vs. −0.003 s). Peak vGRF bias also decreased from −0.310 BW to 0.159 BW ( p<0.01 ; Cohen’s d = 1.45). Furthermore, error standard deviations were reduced across all metrics.

**Conclusion:**

This study validates an IMU framework improving running gait detection. Through sensor fusion, MFD-GED enables high-fidelity parameter estimation. While lab-validated for healthy young males, findings affirm its potential running for future gait monitoring tasks, aiming to offer a reliable tool for professionals in the field.

## 1 Introduction

Quantitative gait analysis, a sophisticated method for evaluating human locomotion, serves dual purposes in clinical and sports settings: as a crucial clinical tool for injury prevention and as a performance optimization mechanism for athletes (Wang et al., 2003; Tao et al., 2012; Chen et al., 2012; Sethi et al., 2022). Key gait spatiotemporal and kinetic parameters, such as stride length, step frequency, contact time, flight time, and ground reaction force, are essential for evaluating running injury (Nijs et al., 2022; Watanabe et al., 2023; Mo and Chow, 2018) and performance (Napier et al., 2015; Di Michele and Merni, 2014). Traditionally, gait assessment relies on subjective clinical observations or rating scales, which are often insufficient for detecting subtle changes caused by training, fatigue, or injury (Ferber and Macdonald, 2014). Although gold-standard laboratory methods, such as optical motion capture systems, force plates, and instrumented walkways, offer precise measurements (Muro-De-La-Herran et al., 2014), they require expensive equipment, dedicated spaces, and complex postprocessing, often delaying access to results and decision-making. These limitations hinder their practicality in routine clinical use or real-world sports environments, where capturing biomechanics during actual running or walking is crucial (Norris et al., 2014).

Advances in wearable technology, particularly improvements in the accuracy, sensitivity, and algorithms of inertial measurement units (IMUs), now enable gait measurement in free-living conditions (Tao et al., 2012), enhancing the effectiveness of IMU-based gait analysis systems for evaluating walking and running biomechanics (Reenalda et al., 2019). These wearable IMUs, consisting of tri-axial accelerometers and gyroscopes, enable the computation of specific gait variables and offer a practical alternative because of their compactness and cost-effectiveness (Mason et al., 2023b; Stuart et al., 2021). The accelerations and angular velocities recorded by body-mounted IMUs enable estimation of running temporal, kinematic and kinetic parameters. As running is cyclic, precise identification of key gait events is essential for partitioning continuous data into discrete gait cycles and deriving reliable, widely reported biomechanical and performance descriptors. Evidence from controlled experiments has revealed strong correlations between distinct signal characteristics, such as extrema in angular-velocity signals, and specific gait events, including initial contact (IC), terminal contact (TC), mid-stance (MS), and mid-swing (MSW) (Mariani et al., 2013; Lee and Park, 2011). Several IMU-based methods identify IC and TC events using sensors affixed to the pelvis (Bergamini et al., 2012), shanks (Yang et al., 2022) or feet (Falbriard et al., 2018). In contrast, foot-mounted units consistently yield the most accurate spatiotemporal estimates (Zrenner et al., 2020). Since lower-trunk and shank placements often rely on semi-elastic belts that can slip or detach under running impact forces, compromising the accuracy of derived gait parameters (Chew et al., 2018).

Common approaches for gait event detection from foot-mounted IMUs include rule-based algorithms (Prasanth et al., 2021) like the angular-velocity-based gait-segmentation (AVGS) method (Luo et al., 2024; Fadillioglu et al., 2020), template-based techniques using dynamic time warping (DTW) (Wang et al., 2016), and machine learning methods (Zhang et al., 2019). The latter two approaches, while sophisticated, often lack generalizability, as their performance can be constrained by the subject-specific datasets used for template construction or model training. In contrast, rule-based methods such as AVGS are computationally efficient and widely deployed, but their accuracy is highly sensitive to variations in foot-strike pattern and running speed (Mitschke et al., 2017). For instance, defining IC based on the angular velocity minimum causes the detected event timing to shift progressively earlier as the strike pattern transitions from rearfoot to forefoot, compromising reliability (Mitschke et al., 2017). Similarly, TC detection exhibits a systematic, speed-dependent bias, as changes in running speed alter the underlying kinematic signatures and reduce accuracy (Yang et al., 2022; Falbriard et al., 2018).

Beyond the accurate detection of IC and TC, identifying the MS phase is also critical, particularly for estimating spatial parameters. The MS event is integral to the zero-velocity update (ZUPT) technique, a widely used method for correcting the integral drift that arises when calculating displacement from acceleration data. To identify this stationary phase, many studies employ detectors based on angular rate energy (ARE) (Li and Wang, 2014; Zhang et al., 2017), while others utilize methods such as acceleration moving variance (MV) to analyze signals from both the accelerometer and gyroscope for robust MS identification (Luo et al., 2024). Notably, these studies were based on fixed thresholds or windows, potentially limiting their robustness across different gait patterns and running speeds.

This limitation highlights the need for more adaptive and refined event-detection algorithms to achieve high-accuracy parameter estimation in running gait analysis. To address this, this study introduces a multi-sensor fusion with dynamic gait event detection (MFD-GED) framework. This refined approach is specifically designed to improve the precision of gait event identification and, consequently, deliver more accurate and reliable gait metrics. Leveraging the cyclical nature of running and the periodic structure of inertial signals, the MFD-GED framework integrates multi-sensor fusion of acceleration and angular-velocity features with a parametric event-detection strategy to improve estimation precision. The method extracts eight gait spatiotemporal and kinetic parameters, including stride velocity (SV), stride length (SL), step frequency (SF), stride time (ST), contact time (CT), swing time (SWT), flight time (FT), and peak vertical ground reaction force (vGRF). Then, the concurrent validity of the proposed MFD-GED method was evaluated during a 10-m run test (10MRT). Performance was assessed against two benchmarks: a laboratory reference system (LAB), composed of optical motion capture and three force plates, and the conventional AVGS method, which served as a point of comparison for accuracy. Ultimately, this study aims to demonstrate that the proposed MFD-GED framework enables single foot-mounted IMU to provide gait parameters with an accuracy comparable to laboratory systems, while offering improved robustness over conventional algorithms in real-world running scenarios.

## 2 Materials and methods

### 2.1 Algorithm description

#### 2.1.1 Preprocessing

Mounting the IMU at the instep resulted in only a rough alignment with the foot coordinate system. To clarify the sensor orientation, the sensor coordinate system was defined as follows: the x-axis was aligned with the medial-lateral direction, the y-axis with the anterior-posterior direction, and the z-axis with the vertical direction. To establish an initial orientation reference for aligning the body frame with the sensor frame, the gravity vector expressed in the sensor frame was estimated by averaging tri-axial acceleration data recorded during periods of minimal movement, identified as intervals where the angular velocity remained below 0.0436 rad/s. To estimate the gravity direction in the sensor (body) frame, the global gravity vector was rotated using the current orientation quaternion $q=\begin{array}{ccc} \left[q_{0}\right. & q_{1} & \begin{array}{cc} q_{2} & \left.q_{3}\right] \end{array} \end{array}$ . The rotation matrix from navigation frame to sensor frame $C_{n}^{s}$ was defined as Equation 1.

$$C_{n}^{s}=\left[\begin{array}{ccc} 1-2q_{2}^{2}-2q_{3}^{2} & 2\left(q_{1}q_{2}+q_{3}q_{4}\right) & 2\left(q_{1}q_{3}-q_{2}q_{4}\right)\\ 2\left(q_{1}q_{2}-q_{3}q_{4}\right) & 1-2q_{1}^{2}-2q_{3}^{2} & 2\left(q_{2}q_{3}+q_{1}q_{4}\right)\\ 2\left(q_{1}q_{3}+q_{2}q_{4}\right) & 2\left(q_{2}q_{3}-q_{1}q_{4}\right) & 1-2q_{1}^{2}-2q_{2}^{2} \end{array}\right]$$ (1)

At each subsequent time step, a parametric correction based on the Mahony complementary filter was employed to compensate for gyroscope drift (Kim et al., 2015). The estimated gravity direction $g^{s}$ in the body (sensor) frame was computed by transforming the global gravity vector via the current rotation matrix $C_{n}^{s}$ , where ‘n’ denotes the navigation frame (e.g., global frame) and ‘s’ denotes the sensor frame. Then the expected gravity vector $g^{s}$ in the sensor frame was computed as Equation 2.

$$g^{s}=\left[\begin{array}{c} g_{x}\\ g_{y}\\ g_{z} \end{array}\right]=C_{n}^{s}\left[\begin{array}{c} 0\\ 0\\ g \end{array}\right]$$ (2)

where $g^{s}$ is the estimated gravity vector in the sensor frame, and g is gravitational acceleration.

This transformed gravity estimate was then compared with the measured acceleration vector to obtain an error vector e via a cross-product. The error vector $e={\begin{array}{ccc} \left[e_{x}\right. & e_{y} & \left.e_{z}\right] \end{array}}^{T}$ was defined as the cross product between the measured acceleration $a^{s}$ and the estimated gravity vector as Equation 3.

$$e=a^{s}\times g^{s}=\left[\begin{array}{c} e_{x}\\ e_{y}\\ e_{z} \end{array}\right]$$ (3)

where e is the error vector obtained via the cross product of the measured acceleration $a^{s}={\begin{array}{ccc} \left[a_{x}\right. & a_{y} & \left.a_{z}\right] \end{array}}^{T}$ and estimated gravity vector $g^{s}$ .

A proportional-integral correction framework was applied to update the gyroscope signals using this error. The corrected angular velocity is computed as Equation 4.

$$\left\{\begin{array}{c} \omega_{x,corr}=\omega_{x}+K_{p}\cdot e_{x}+K_{i}\int e_{x}dt\\ \omega_{y,corr}=\omega_{y}+K_{p}\cdot e_{y}+K_{i}\int e_{y}dt\\ \omega_{z,corr}=\omega_{z}+K_{p}\cdot e_{z}+K_{i}\int e_{z}dt \end{array}\right.$$ (4)

where $\omega_{x,corr}$ , $\omega_{y,corr}$ , $\omega_{z,corr}$ are the corrected angular velocity velocities; $K_{p}$ and $K_{i}$ are proportional and integral gains; $e_{x}$ , $e_{y}$ , $e_{z}$ are the components of the error vector.

The orientation quaternion is updated using a second-order approximation as Equation 5.

$$\left[\begin{array}{c} q_{0}^{\prime }\\ q_{1}^{\prime }\\ \begin{array}{c} q_{2}^{\prime }\\ q_{3}^{\prime } \end{array} \end{array}\right]=\left[\begin{array}{cccc} 1 & -\frac{\omega_{x}}{2}△t & -\frac{\omega_{y}}{2}△t & -\frac{\omega_{z}}{2}△t\\ \frac{\omega_{x}}{2}△t & 1 & \frac{\omega_{z}}{2}△t & -\frac{\omega_{y}}{2}△t\\ \frac{\omega_{y}}{2}△t & -\frac{\omega_{z}}{2}△t & 1 & \frac{\omega_{x}}{2}△t\\ \frac{\omega_{z}}{2}△t & \frac{\omega_{y}}{2}△t & -\frac{\omega_{x}}{2}△t & 1 \end{array}\right]\cdot \left[\begin{array}{c} q_{0}\\ q_{1}\\ \begin{array}{c} q_{2}\\ q_{3} \end{array} \end{array}\right]$$ (5)

where $q_{0}^{\prime }$ , $q_{1}^{\prime }$ , $q_{2}^{\prime }$ , $q_{3}^{\prime }$ are the updated quaternion components; $\omega_{x}$ , $\omega_{y}$ , $\omega_{z}$ are the corrected angular velocities; and Δt is the time step interval.

**FIGURE 1:**
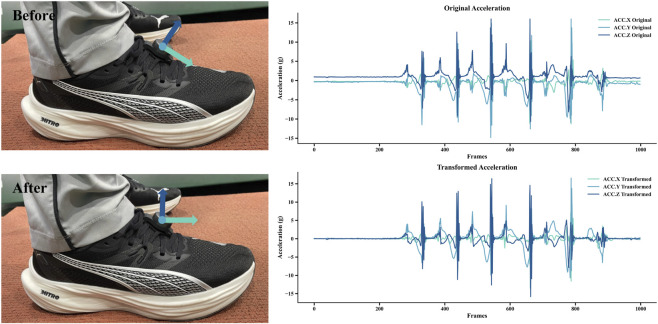
Foot-mounted IMU placement (left) and comparison of triaxial acceleration signals (right) before and after coordinate transformation. The top panel shows the raw acceleration in the sensor frame, whereas the bottom panel displays the transformed acceleration in the navigation (global) frame. During stationary phases, the transformed acceleration signals stabilize near 0 g along each axis, confirming proper alignment with the navigation (global) frame.

The corrected angular velocity was subsequently used to incrementally update the quaternion representation of orientation via a second-order approximation. Finally, the raw acceleration data in the sensor frame were first rotated into the global frame via quaternion-based transformation $a^{s}\left(t\right)=C_{n}^{s}\left(t\right)\cdot a^{n}\left(t\right)$ , and then the gravitational component was subtracted from the vertical axis of the rotated acceleration vector (Figure 1). The final corrected acceleration was computed as Equation 6.

$$a^{s}\left(t\right)=C_{n}^{s}\left(t\right)\cdot a^{n}\left(t\right)-g^{n}$$ (6)

where $a^{s}\left(t\right)$ is the raw acceleration in the sensor frame, $C_{n}^{s}\left(t\right)$ is the rotation matrix from sensor to navigation frame, $g^{n}={\begin{array}{ccc} \left[0\right. & 0 & \left.g\right] \end{array}}^{T}$ is the gravity vector in the navigation frame, and $a^{n}\left(t\right)$ is the gravity-compensated acceleration used for integration.

#### 2.1.2 Gait event detection

Gait is characterized as a stable, quasi-periodic cyclic phenomenon that defines the pattern of bipedal locomotion (Lu et al., 2023). This inherent periodicity manifests in inertial sensor data as quasi-periodic waveforms, which exhibit approximate regularity but possess cycle durations that vary within a specific range. To mitigate noise, both angular velocity and acceleration signals were processed using a fourth-order Butterworth low-pass filter with a 6 Hz cutoff frequency (Hutabarat et al., 2020). Subsequently, gait cycle segmentation was performed using the sagittal-plane angular velocity ( $\omega_{x}$ ), as running occurs predominantly in this plane. The local maxima of the $\omega_{x}$ signal were identified as MSW candidates. However, given the varying cycle lengths inherent in quasi-periodic signals, fixed thresholds are often insufficient. Therefore, to robustly validate these candidates, an adaptive threshold approach was adopted (Prigent et al., 2023). Specifically, an adaptive threshold was established from the standard deviation of angular velocity signal, which served as an estimate of the gyroscope noise level. Then, positive peaks exceeding this dynamic threshold were confirmed as MSW events, with each gait cycle defined as the interval between two consecutive MSWs.

Following gait segmentation, the traditional AVGS algorithm identifies the IC as the negative peak corresponding to the rapid deceleration just before foot strike (Ruiz-Ruiz et al., 2024). A common refinement of this technique employs a zero-crossing rule, which designates IC as the first instant that angular velocity transitions from positive to non-positive (Luo et al., 2024). To build upon these methods and enhance event detection robustness, we introduced the MFD-GED framework. This proposed methodology refines the temporal localization of IC and TC by strategically fusing kinematic and kinetic definitions derived from gyroscope and accelerometer data.

For IC detection, a coarse estimation is initially established using the AVGS method. To enhance precision, a stride-adaptive search window, calculated as 30% of the total stride duration, is centered on this preliminary timestamp. Within this localized temporal domain, the algorithm seeks to reconcile two distinct biomechanical markers: the kinematic marker, defined as the zero-crossing in sagittal angular velocity ( $\omega_{x}$ ) representing the rotational transition from swing to stance; and the kinetic marker, defined as the distinct peak in anterior-posterior acceleration ( $a_{ap}$ ) corresponding to the instantaneous braking force upon foot strike. Physically, these two markers are temporally non-aligned. The high-frequency response of the accelerometer captures the abrupt impact transient (kinetic event) with higher temporal resolution, whereas the angular velocity (kinematic event) provides a stable definition of the rotational phase. To resolve this discrepancy, the algorithm computes the final IC timestamp through a weighted linear combination of these two candidates. Consequently, a dominant weight is assigned to the acceleration peak to align the event with the precise moment of impact, while the gyroscope data is utilized to constrain detection errors.

**FIGURE 2:**
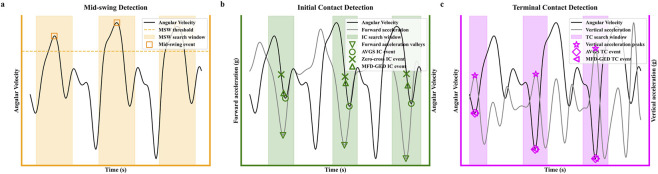
The proposed gait event detection pipeline using data from foot-mounted IMU. **(a)** Mid-swing (MSW) detection based on identifying angular velocity peaks that exceed a dynamic threshold. **(b)** Initial Contact (IC) detection, comparing the outputs of the conventional AVGS, the zero-crossing, and the proposed MFD-GED method. **(c)** Terminal Contact (TC) detection, comparing the AVGS method with the MFD-GED method. Shaded regions indicate the dynamic search windows for each event.

Subsequent to IC identification, the TC event is determined using a similar sensor fusion strategy within a dynamic forward-projected window. The algorithm isolates the kinematic signature of the toe-off mechanism (inflection point in angular velocity) and the corresponding kinetic marker (propulsive peak in acceleration). Recognizing that toe-off involves both rotational kinematics and propulsive kinetics, the final TC timestamp is computed as a weighted average of these two sensor-derived candidates. This differential weighting strategy leverages specific physical characteristics, including the temporal sharpness of the kinetic signal and the phase stability of the kinematic signal, to determine the gait event timestamps (as illustrated in Figure 2).

#### 2.1.3 Zero-velocity detection for segmentation

Building on (Luo et al., 2024), which utilized angular velocity energy and horizontal acceleration variance detectors for MS detection, we introduced a parametric windowing method to improve the detection accuracy. Specifically, for each interval between two consecutive IC and TC events, the first and last 10% of the stance phase were excluded, considering the low probability of mid-stance occurrence in these regions. Within the remaining portion, a sliding window with a size equal to 30% of the stance phase was applied to compute two key metrics: 1) the mean angular velocity energy across the tri-axial gyroscope signals and 2) the variance of horizontal-plane acceleration. The timestamp subsequently corresponds to the minimum angular velocity energy $t_{\omega }$ , and the timing of the minimum acceleration variance $t_{v}$ is identified. To balance these two indicators, a dynamic weight w was computed as Equation 7.

$$w=\frac{\sigma_{a}^{2}}{\sigma_{a}^{2}+\left|\sigma_{a}^{2}-\sigma_{a,prev}^{2}\right|}$$ (7)

where $\sigma_{a}^{2}$ is the current acceleration variance and where $\sigma_{a,prev}^{2}$ is the variance from the previous MS detection. The final MS timestamp ( $t_{MS}$ ) was obtained via interpolation as Equation 8.

$$t_{MS}=w\cdot t_{\omega }+\left(1-w\right)\cdot t_{v}$$ (8)

**FIGURE 3:**
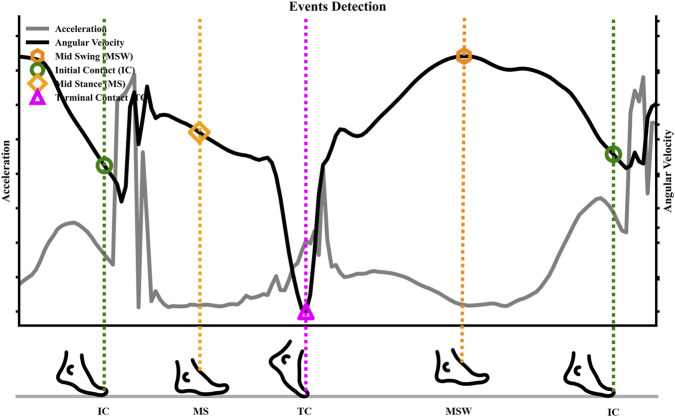
Identification of key gait events within a single running stride using characteristic signals from foot-mounted IMU. The angular velocity (black line) and acceleration (gray line) profiles are used to detect Initial Contact (IC), Mid-Stance (MS), Terminal Contact (TC), and Mid-Swing (MSW). The timing of each event is indicated by a vertical dashed line, with the corresponding foot posture illustrated by the icons below.

This method combines angular velocity energy and acceleration variance in an adaptive framework to improve the robustness of MS detection. Figure 3 shows the IC, MS, TC, and MSW events within a single gait cycle.

### 2.2 Gait parameter estimation

A complete gait cycle comprises two primary phases: stance and swing (Dugan and Bhat, 2005). Following the identification of IC and TC events, temporal parameters for each cycle are calculated directly from these event timing. These event timestamps were subsequently used to calculate the following temporal parameters, including stride time (ST), contact time (CT), and swing time (SWT). ST, CT, and SWT were calculated as the time from foot strike to foot strike of the same foot, from foot strike to terminal contact of the same foot, from terminal contact to foot strike of the same foot, and from terminal contact of one foot to foot strike of the contralateral foot (Patoz et al., 2022), respectively (see Equation 9).

$$\left\{\begin{array}{c} t_{stride\;time}=t_{IC,i+1}-t_{IC,i}\\ t_{contact\;time}=t_{TC,i}-t_{IC,i}\\ t_{swing\;time}=t_{IC,i+1}-t_{TC,i} \end{array}\right.$$ (9)

As the analysis was based on a single foot-mounted IMU, only unilateral gait parameters could be determined. The flight time for the instrumented foot was therefore calculated as Equation 10.

$$t_{flight\;time}=\left(t_{swing\;time}-t_{contact\;time}\right)/2$$ (10)

Stride frequency was calculated from the stride time as Equation 11.

$$t_{stride\;frequnecy}=120/t_{stride\;time}$$ (11)

**FIGURE 4:**
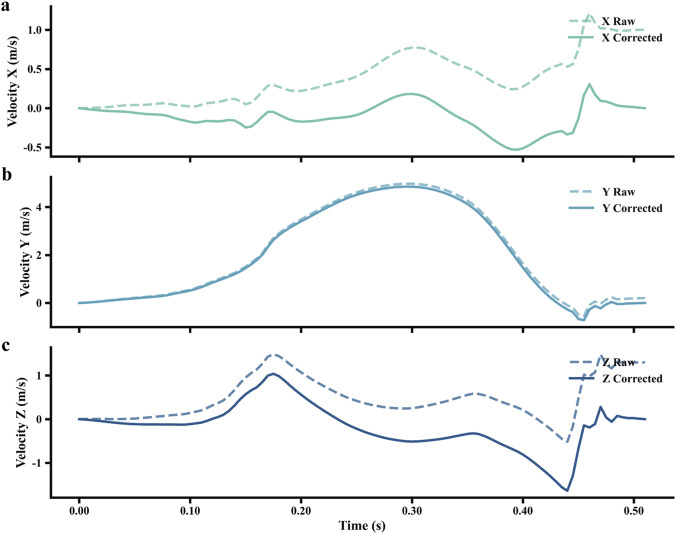
Effect of the drift-correction algorithm on the three-dimensional velocity profile of the foot over a single gait cycle. The panels display the velocity components in the **(a)** X, **(b)** Y, and **(c)** Z directions. Dashed lines represent the raw velocity obtained from direct integration of acceleration data, which exhibits significant drift error. Solid lines show the corrected velocity.

The stride length (SL) for each gait cycle was estimated via double integration of acceleration data. Prior to this process, the acceleration signal was transformed from the sensor’s local coordinate system to a global reference frame to nullify the effects of the foot’s changing orientation. The integration was then performed over the interval between consecutive MSW events, yielding velocity after the first integration and the displacement corresponding to SL after the second. However, direct integration may introduce drift, necessitating a correction method. A zero-velocity updating (ZUPT) method was used to mitigate integral drift (Foxlin, 2005), which assumes zero initial acceleration and velocity at each MS point. Preliminary integration was performed to obtain raw velocities, followed by linear drift correction on the basis of the method in (Luo et al., 2024). The endpoint velocity error was linearly distributed across each stride via a weighting function $w\left(j\right)=j/n$ , ensuring zero-velocity enforcement at both ends of each gait cycle. The corrected velocities were then integrated to obtain the displacement profiles. The individual steps of the velocity correction process are visualized for one sample stride in Figure 4.

Then, the SL ( $d_{stride}$ ) for each gait cycle was computed by integrating the corrected velocity over time and calculating the resultant displacement in the horizontal plane (x- and y-directions, as seen in Equation 12). SV ( $v_{stride}$ ) was then obtained by dividing the SL by the corresponding ST (see Equation 13).

$$d_{stride}=\sqrt{d_{x}{\left[t_{MS,i+1}\right]}^{2}+d_{y}{\left[t_{MS,i+1}\right]}^{2}}$$ (12)

$$v_{stride}=\frac{d_{stride}}{t_{stride}}$$ (13)

**FIGURE 5:**
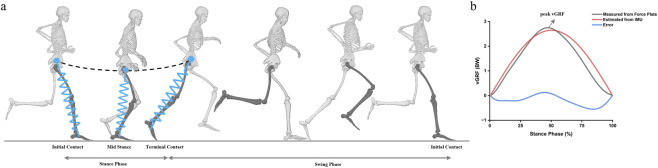
**(a)** The spring-loaded inverted pendulum (SLIP) is used as the spring-mass model of running. **(b)** Measured (solid black line) and estimated (solid red line) vertical ground reaction force‒time waveforms for a single stance phase in one participant. The blue line represents the error, calculated as the discrepancy between the measured and estimated vGRF values. All forces were normalized to body weight (BW).

During running, the musculoskeletal system alternately stores and releases elastic energy, enabling the legs to behave like mass-loaded springs. This principle underlies the spring-loaded inverted pendulum (SLIP) model (McMahon and Cheng, 1990), which serves as a biomechanical template for running. The SLIP framework, commonly referred to as the spring-mass model, represents the runner as a point mass atop a single linear spring leg, as illustrated in Figure 5. A sinusoidal function was used to approximate the vertical ground reaction force (vGRF) waveform (Morin et al., 2005), enabling the estimation of the peak vGRF from CT ( $t_{contact\;time,i}$ ) and FT ( $t_{flight\;time,i}$ ), as Equation 14.

$$F\left(t\right)=\frac{\pi }{2}mg\left(\frac{t_{flight\;time,i}}{t_{contact\;time,i}}+1\right)\text{si}n\left(\frac{\pi }{t_{contact\;time,i}}t\right)$$ (14)

where m is the participant’s body mass and where g is the acceleration due to gravity. The modeled peak vGRF during stance is then derived as Equation 15.

$$F_{\max}=\frac{\pi }{2}\left(\frac{t_{flight\;time,i}}{t_{contact\;time,i}}+1\right)$$ (15)

### 2.3 Experimental setup and protocol

Fifteen healthy male participants from Beijing Sport University (aged 24.07 ± 1.14 years; body height: 177 ± 6.25 cm; body mass: 75.21 ± 8.02 kg; four forefoot strikes and eleven non-forefoot strike runners) voluntarily participated in this study. We recruited only male participants to control for gender variability in gait (Sardroodian and Hosseinzadeh, 2020). The study was conducted in accordance with the Declaration of Helsinki and approved by the Ethics Committee of Beijing Sport University (protocol code: 2024466H).

**FIGURE 6:**
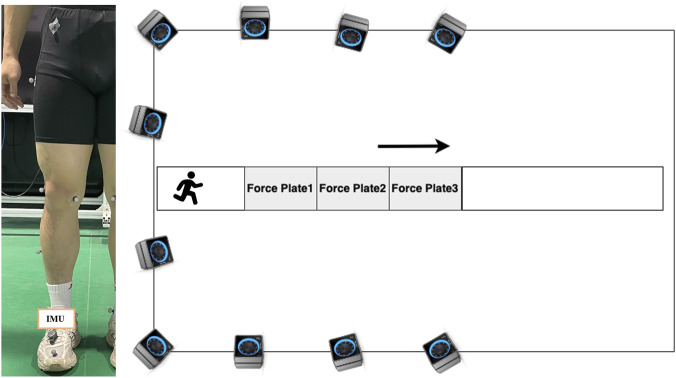
Standardized reference calibration pose setup, placement of reflective markers, IMU on a participant, and layout of the experimental site.

During this study, participants wore shoes fitted with a single 9-axis IMU (STAG, Huawei^®^, Guangdong, China) securely attached to the right instep. To ensure rigid attachment and minimize motion artifacts, the device was secured via a specialized locking foot mount. This mounting mechanism features a hinged clamping base that was inserted beneath two rows of crossed shoelaces and mechanically locked to create a stable anchor point. The sensor unit was then rigidly fastened to the base via a twist-lock interface. The IMU recorded at a sampling rate of 200Hz, and was configured with an accelerometer and a gyroscope range of ±16 g and ±32 rad/s, respectively. During the running test, acceleration and angular velocity data were recorded and transmitted via Bluetooth 5.0 to a mobile phone via the data collection app. A ten-camera high-speed optical motion capture laboratory system (OptiTrack, Natural Point, Corvallis, OR, United States) and three force plates (Kistler, Kistler Instrument Corp.) were used to collect marker data and ground reaction forces (GRFs) simultaneously at 200 Hz and 1,000 Hz, respectively. A total of 19 reflective markers (14 mm diameter) were affixed to specific anatomical landmarks following the OptiTrack Helen Hayes lower-limb marker set protocol. The sites included the sacrum, bilateral anterior superior iliac spines, lateral aspects of the thighs, both lateral and medial knee epicondyles, lateral calves, both lateral and medial malleoli, and the heel and second metatarsal, as shown in Figure 6.

To verify the accuracy of the estimated gait parameters, the study employed a 10-m run test (10MRT) experimental approach (Scivoletto et al., 2011; Castro-Piñero et al., 2023). The 10MRT was selected as it allows participants to achieve a consistent, self-selected running speed, thereby facilitating the capture of representative steady-state gait cycles for biomechanical analysis. Specifically, the concurrent validity of the wearable sensor was evaluated via the LAB system. The participants performed three trials of the 10MRT on a 14-m laboratory track at a self-selected comfortable speed. For each trial, one complete gait cycle with a valid foot strike was captured. Collecting three valid trials aligns with established protocols for gait analysis, ensuring sufficient test-retest reliability for the estimated parameters (Cheng et al., 2020; Santos et al., 2025). Data were simultaneously acquired from both the LAB system and the IMU during these trials.

The marker positions and GRF data collected by the LAB system were filtered via Butterworth fourth-order low-pass filters with cutoff frequencies of 7 Hz (Maiwald et al., 2009) and 25 Hz (Tan et al., 2019), respectively. IC and TC events were identified via a 25 N threshold of vGRF (Hoogkamer et al., 2018). We segment the data collected from the LAB system into individual strides and extract the corresponding gait parameters for comparison with the estimated results obtained from the IMU. Each trial required the participant to target the force plate with the right foot, resulting in three complete gait cycles for comparison. Average measurements were generated as a unified reference for comparison.

### 2.4 Statistical analysis

Means and SDs were calculated for each gait parameter for each of the two systems over the three running trials. Gait parameter normality was tested via the Shapiro–Wilk test, with p>0.05 indicating normality. To assess validity, the following methods were subsequently employed on the basis of their comparison with the LAB reference system: Pearson correlation coefficients (Pearson’s r) and intraclass correlation coefficients (ICC_3,k_) for the mean of k-ratings (two-way mixed model) were subsequently used to determine the level of agreement. The relationship between the LAB reference system and the IMU-based method was analyzed through a linear regression model. Furthermore, Bland‒Altman plots of bias and limits of agreement (LoA) were used to assess the magnitude of the agreement (Bland and Altman, 1986). Additionally, algorithm performance was evaluated by quantifying timing errors between force-based reference events (rather than marker-based events) and algorithm-predicted events. Agreement in timing was assessed using the mean timing error, its 95% confidence interval (CI), and the mean absolute error (MAE). CIs were calculated as $\bar{x}\pm 1.96s\bar{x}$ , where $s\bar{x}$ denotes the standard error of the mean. To statistically compare the performance differences between the proposed MFD-GED method and the conventional AVGS method, paired-sample t-tests were conducted on the systematic bias of estimated parameters.

Pearson’s r and ICC values below 0.50 were classified as poor, values between 0.51 and 0.75 as moderate, values between 0.76 and 0.90 as good, and values between 0.91 and 1.00 as excellent (Koo and Li, 2016). Furthermore, the magnitude of the significant changes was analyzed using Cohen’s effect sizes: small effect (d ≥ 0.2), medium effect (d ≥ 0.5), and large effect (d ≥ 0.8) (Cohen, 2013). Statistical significance was set at p < 0.05. Statistical procedures were conducted via SPSS software (v26.0, IBM, Armonk, NY, United States).

## 3 Results

The gait data of fifteen participants performing running tasks resulted in 360 gait measurements for analysis (15 participants × 8 outcomes × 3 repeats). Normality tests (Shapiro–Wilk) confirmed that gait data from both the single foot-mounted IMU (MFD-GED) and the laboratory system (LAB) followed a normal distribution ( p>0.05 ).

**FIGURE 7:**
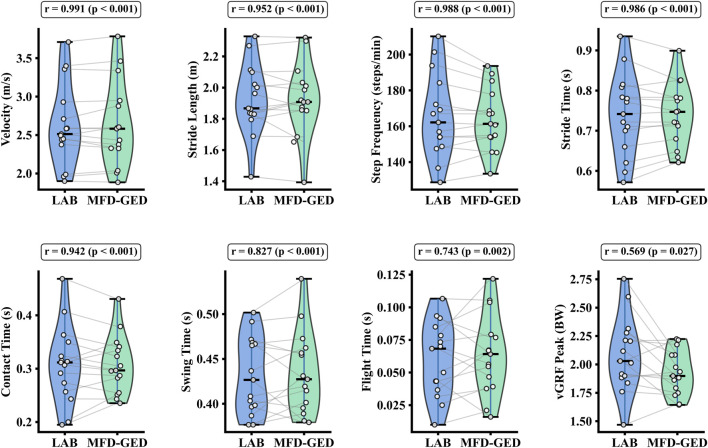
Violin plots comparing eight spatiotemporal and kinetic gait parameters from the LAB system and the proposed MFD-GED method. Gray lines connect paired measurements from individual trials, and the Pearson’s r values for each parameter is displayed above the corresponding panel.

**FIGURE 8:**
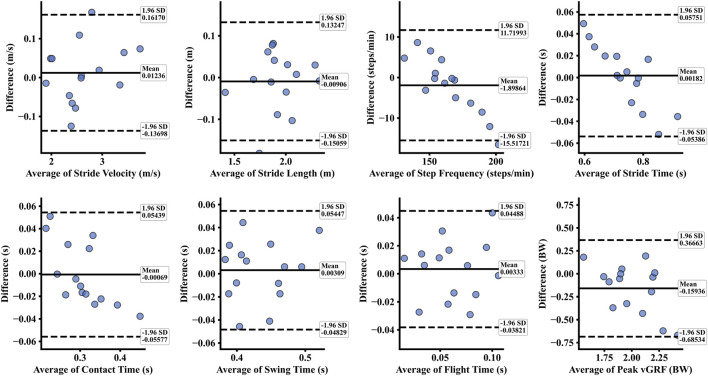
Bland-Altman analysis of agreement between the proposed MFD-GED method and the LAB system for eight spatiotemporal and kinetic gait parameters. In each plot, the solid line indicates the mean difference (bias), and the dashed lines represent the 95% limits of agreement.

The MFD-GED method demonstrated high concurrent validity with the LAB standards across all measured parameters. As detailed in Table 1, the estimation errors were minimal; specifically, the mean difference for CT was 0.001 ± 0.028 s, and for SL was 0.009 ± 0.072 m. High consistency was further evidenced by the correlation analysis (Table 2), where basic spatiotemporal parameters (SV, SL, SF, ST, CT) achieved correlation coefficients greater than 0.94 and ICCs above 0.90. While the kinetic parameter (peak vGRF) showed a moderate correlation (r = 0.569), the distributional analysis confirms that the MFD-GED method effectively captures the data range and density of the LAB reference (see Figure 7). The Bland-Altman analysis indicated robust agreement, with the majority of data points falling within the 95% LoA (see Figure 8).

**TABLE 1:** Estimation errors of gait parameters in 10MRT tests.

Parameters	Error (LAB –MFD-GED)			
	Mean	SD	MAE	95% LoA
SV (m/s)	−0.012	0.076	0.059	−0.162∼0.137
SL (m)	0.009	0.072	0.053	−0.132∼0.151
SF (steps/min)	1.899	6.948	5.296	−11.720∼15.517
ST (s)	−0.002	0.028	0.022	−0.058∼0.054
CT (s)	0.001	0.028	0.024	−0.054∼0.056
SWT (s)	−0.003	0.026	0.021	−0.054∼-0.048
FT (s)	−0.003	0.021	0.018	−0.045∼0.038
Peak vGRF (BW)	0.159	0.268	0.218	−0.367∼0.685

**TABLE 2:** Descriptive statistics of gait parameters obtained from different methods (i.e., the LAB system and MFD-GED method) in the running protocols and the validity of the IMU method compared with the LAB method (i.e., Pearson’s r and ICCs).

Parameters	LAB	MFD-GED		
	Mean (SD)	Mean (SD)	Pearson’s r	ICC_3,1_
SV (m/s)	2.626 ± 0.531	2.639 ± 0.553	0.991	0.990
SL (m)	1.929 ± 0.226	1.920 ± 0.236	0.952	0.951
SF (steps/min)	165.752 ± 23.363	163.853 ± 17.171	0.988	0.943
ST (s)	0.740 ± 0.102	0.741 ± 0.077	0.986	0.951
CT (s)	0.308 ± 0.072	0.307 ± 0.054	0.942	0.903
SWT (s)	0.432 ± 0.043	0.435 ± 0.046	0.827	0.825
FT (s)	0.060 ± 0.028	0.064 ± 0.030	0.743	0.741
Peak vGRF (BW)	2.086 ± 0.326	1.926 ± 0.196	0.569	0.502

**FIGURE 9:**
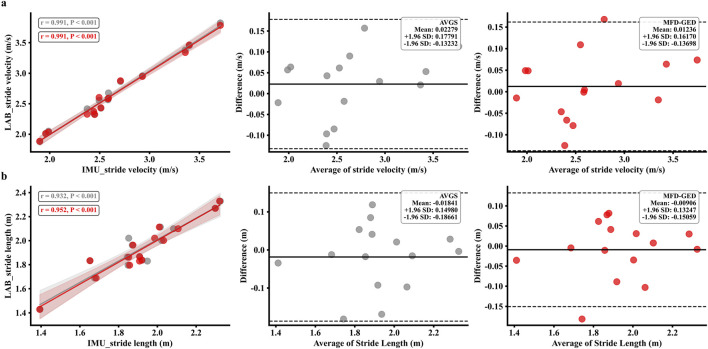
Validation of **(a)** SV and **(b)** SL estimation, comparing the MFD-GED method (red) with AVGS method (gray) against the LAB system. The Pearson correlation and Bland–Altman plots collectively demonstrate that the MFD-GED method yields superior agreement with the standard, as indicated by stronger correlation, reduced measurement bias, and narrower limits of agreement.

**FIGURE 10:**
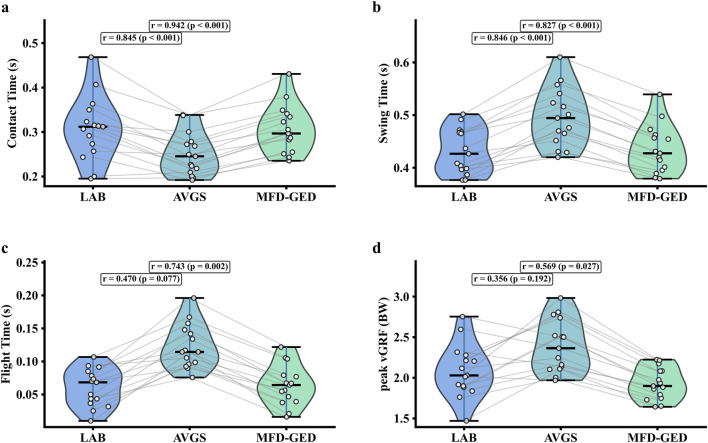
Violin plots comparing gait parameter distributions from the LAB, AVGS, and MFD-GED method. Panels display **(a)** contact time, **(b)** swing time, **(c)** flight time, and **(d)** peak vertical ground reaction force (vGRF). Gray lines connect paired measurements from the same trial, and the corresponding Pearson’s r values.

**FIGURE 11:**
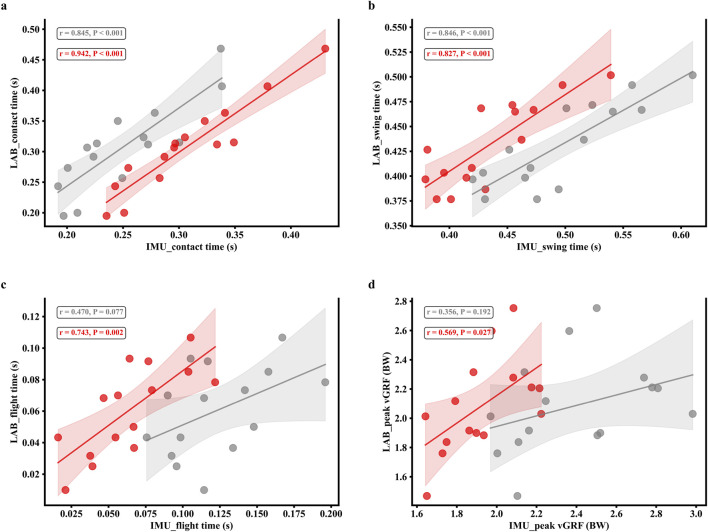
Linear regression analysis of gait parameters estimated by the proposed MFD-GED method (red) and AVGS method (gray), plotted against the LAB. The four panels compare results for **(a)** contact time, **(b)** swing time, **(c)** flight time, and **(d)** peak vertical ground reaction force (vGRF). Shaded areas represent the 95% confidence intervals, and the corresponding Pearson’s r values.

Comparing the proposed MFD-GED algorithm with the conventional AVGS method revealed significant improvements in estimation accuracy. As summarized in Table 3, the MFD-GED method yielded consistently lower systematic bias and random error. For spatial parameters, the reduction in bias was not statistically significant ( p>0.05 ). Specifically, the mean bias for CT was notably reduced to 0.001 ± 0.028 s, compared to the overestimate of 0.057 ± 0.041 s with the AVGS method ( p<0.01 ; Cohen’s d = 1.62). Significant bias reductions were also achieved for SWT (−0.003 ± 0.026 s vs. −0.065 ± 0.030 s; p<0.01 ; Cohen’s d = 2.20) and FT (−0.003 ± 0.021 s vs. −0.063 ± 0.032 s; p<0.01 ; Cohen’s d = 2.18). Similarly, for peak vGRF, the MFD-GED method mitigated the underestimation bias found in AVGS, improving the mean error from −0.310 BW to 0.159 BW ( p<0.01 ; Cohen’s d = 1.45). These quantitative improvements are corroborated by the graphical analyses. The MFD-GED estimates showed tighter alignment with the identity line for spatial parameters, whereas the AVGS estimates exhibited wider dispersion and outliers (see Figure 9). Furthermore, the distributional characteristics of the MFD-GED parameters closely mirrored the LAB reference, while the AVGS method resulted in marked deviations, particularly for Contact Time and peak vGRF (see Figure 10). Regression analysis further confirmed this trend, indicating stronger linearity and narrower confidence intervals for the MFD-GED method compared to the AVGS algorithm (see Figure 11).

**TABLE 3:** Estimation errors of gait parameters via different methods.

Parameters	Error (mean ± SD)	
	AVGS	MFD-GED
SV (m/s)	−0.023 ± 0.079	−0.012 ± 0.076
SL (m)	0.018 ± 0.086	0.009 ± 0.072
SF (steps/min)	3.177 ± 6.951	1.899 ± 6.948
ST (s)	−0.008 ± 0.026	−0.002 ± 0.028
CT (s)	0.057 ± 0.041	0.001 ± 0.028
SWT (s)	−0.065 ± 0.030	−0.003 ± 0.026
FT (s)	−0.063 ± 0.032	−0.003 ± 0.021
Peak vGRF (BW)	−0.310 ± 0.369	0.159 ± 0.268

## 4 Discussion

This study presents a refined method for accurate running gait analysis using a single foot-mounted IMU. To improve event detection, we introduce a fusion-based and parametric method for events identification. The method computes comprehensive spatiotemporal and kinetic gait parameters relevant to running performance assessment. We then evaluated the validity of the proposed method by comparing it with the LAB systems consisting of optical motion capture and force plates during the 10MRT. The results confirm that the MFD-GED method achieves high consistency with the laboratory standards, providing a robust tool for running gait analysis.

The comparative analysis demonstrates that the MFD-GED framework significantly outperforms the conventional AVGS algorithm, particularly in minimizing estimation errors for SV and SL. Specifically, the mean error in SV was reduced from −0.023 ± 0.079 m/s (AVGS) to −0.012 ± 0.076 m/s (MFD-GED), and SL error was reduced from 0.018 ± 0.086 m to 0.009 ± 0.072 m (see Table 3). This performance improvement is fundamentally attributed to the robust detection of the MS phase, which is critical for the effective application of the ZUPT algorithm. Direct integration of acceleration data inevitably leads to signal drift. While the previous method uses a fixed 150 ms window (Luo et al., 2024), whereas the MFD-GED parametric method excludes the initial and terminal 10% and applies a 30% sliding window to allow better adaptation to individual gait patterns and supports more robust MS estimation. These improvements are attributed to more accurate MS detection, which improves ZUPT and subsequent parameter estimation. Precise temporal localization of the MS phase ensures that the ZUPT correction is applied exclusively when the foot is mechanically stationary. By mitigating the integration drift that typically accumulates during velocity and position estimation, the proposed method achieves the superior spatial accuracy. This aligns with prior research has highlighted that accurate stance detection is critical for SV and SL estimation because of the application of ZUPT at these points (Zhao et al., 2016).

Furthermore, the MFD-GED method achieved superior accuracy for temporal parameters, including CT, SWT, and FT demonstrating statistically significant improvements across all metrics ( p<0.01 ). Notably, for CT, our method achieved a negligible mean error of 0.001 ± 0.028 s, contrasting sharply with the overestimate of 0.057 ± 0.041 s observed with the AVGS method. Crucially, the method also significantly improved FT estimation, reducing the mean error from −0.063 s (AVGS) to −0.003 s (MFD-GED). The observed performance enhancements are directly attributable to the proposed multi-sensor fusion strategy. While conventional approaches, such as AVGS, rely predominantly on angular velocity zero-crossings that inherently exhibit systematic latency, the current framework integrates the kinematic stability of the gyroscope with the superior temporal resolution of the accelerometer. By implementing a weighted fusion strategy to reconcile discrepancies between kinetic (impact/propulsion) and kinematic (rotational) signatures, the algorithm effectively eliminates the systematic latency often observed in single-sensor detection (Fadillioglu et al., 2020). Consequently, this precise temporal localization of IC and TC events directly translates to improved accuracy in CT and SWT calculations. As FT in single-sensor setups is derived based on the accurate delimitation of stance and swing phases (assuming gait symmetry), the minimization of errors in the primary events (IC and TC) naturally propagated to the derived FT metric. However, consistent with previous studies, the correlation for FT (r = 0.743) remained slightly inferior to that of CT (r = 0.942). This aligns with findings that single-IMU systems face inherent challenges in FT calculation, as the sensor is mounted on only one foot, requiring FT to be estimated rather than directly measured (Allseits et al., 2017; Trojaniello et al., 2014).

Regarding kinetic parameters, although the consistency of peak vGRF was moderate compared to the other gait parameters (Pearson’s r = 0.569, ICC = 0.502, p<0.05 ). Rather than directly measuring vGRF with force plates, the MFD-GED method estimates it using a spring-mass model of running (Blickhan, 1989). This model-based approach relies on assumptions of a symmetrical sinusoidal force profile and constant leg stiffness, which introduces estimation errors compared to the ground truth. While data-driven approaches like Machine Learning (ML) have demonstrated higher accuracy in vGRF estimation, they often function as ‘black boxes’ and demand extensive training datasets and computational resources, limiting their deplorability on low-power wearable devices (Lin et al., 2025). In contrast, the spring-mass model offers a significant advantage in terms of computational efficiency and physical interpretability, making it highly suitable for real-time applications. Despite its inherent idealizations, this model provides a robust framework for estimating kinetics in scenarios where direct force measurement is impractical. The feasibility of using such model-based estimates has been widely demonstrated in field-based sports research where deploying force plates is not feasible (Hanley et al., 2023). Furthermore, for load monitoring purposes, recent evidence suggests that characterizing trends in cumulative loading is more critical for injury risk assessment than achieving absolute precision in instantaneous magnitude (Ridge et al., 2025).

Bland–Altman analysis revealed a trend where the LAB system recorded slightly larger values for gait parameters (SV, SL, SF, CT) than the MFD-GED method (Figure 8). This observation aligns with the results from other wearable gait analysis systems (Adams et al., 2016; García-Pinillos et al., 2019; Mason et al., 2023a). Furthermore, a proportional bias was observed in certain parameters (e.g., SF, ST, and CT), where the measurement difference varied systematically with the magnitude of the parameter. Two primary factors likely contribute to both the underestimation and this non-uniform error distribution. First, signal processing methodology plays a critical role. As running speed varies (changing the signal frequency content), a fixed filter may attenuate signal peaks and smooth transitions differently across the range of motion (Miller et al., 2022). Consequently, this may result in temporal drift or slight underestimations of event durations. Second, physical coupling artifacts significantly influence measurement fidelity. Even with secure sensor attachment, residual vibrations relative to the anatomical segment, commonly termed soft tissue artifacts, can inherently introduce minor kinematic deviations (Chapman et al., 2019). Crucially, in high-impact activities, faster running speeds generate larger impact forces, intensifying these micro-movements and further contributing to the trends observed in the error distribution. Despite this, Bland–Altman analysis revealed that almost all the data points fell within the 95% LoA, indicating that the MFD-GED method was generally consistent with the LAB reference.

The quantitative superiority of the MFD-GED framework extends to its methodological robustness and translational utility. Regarding algorithmic robustness, the proposed fusion-based strategy demonstrated superior stability compared to the threshold-based AVGS method, as evidenced by the attenuated error dispersion across key temporal metrics (e.g., CT error SD reduced from 0.041 s to 0.028 s). This reduction in variability indicates that the parametric event detection logic maintains speed-invariant stability, effectively mitigating the signal noise and kinematic artifacts that typically degrade the performance of fixed-threshold algorithms at higher running velocities. From a translational perspective, the elimination of systematic bias is critical for clinical and performance applications. The substantial bias observed in the AVGS method (e.g., 0.057 s for CT) poses a risk of masking clinically relevant gait asymmetries or longitudinal adaptations. In contrast, the MFD-GED method achieved near-zero systematic bias (0.001 s), providing the discriminative sensitivity required to detect subtle temporal deviations. This level of precision is a prerequisite for high-fidelity monitoring in sports medicine, enabling the reliable identification of early-stage fatigue markers or injury risks that would otherwise remain undetectable within the error margins of conventional methods (Kim et al., 2025; Zhang et al., 2025).

Effective IMU-based gait analysis depends on signal processing algorithms to convert raw inertial measurements into interpretable gait parameters (Young et al., 2022). Precise detection of IC and TC points is essential for obtaining reliable spatiotemporal metrics (Benson et al., 2019). Our study supports these principles and reinforces the utility of the foot as an optimal location for such high-precision analysis (Uno et al., 2022). While IMUs are often placed on the trunk (Morris et al., 2019) or shanks (Mancini and Horak, 2016), the instep attachment used in this study offers a distinct advantage for directly capturing foot-ground interaction mechanics. Specifically, the MFD-GED method effectively measures running gait parameters with high accuracy, consistent with validation studies of other wearable devices (He et al., 2024; Yoon et al., 2024). This aligns with the current commercial landscape, where systems such as RunScribe™ (Garcia-Pinillos et al., 2020) and Stryd (García-Pinillos et al., 2021), are mounted on the instep, a convenient and secure location ideal for rapid gait assessment and monitoring during running training. These results confirm that with refined event detection algorithms, a single foot-mounted IMU provides a convenient, secure, and robust solution for rapid gait assessment and monitoring in real-world training environments (Yamamoto et al., 2022; Wang et al., 2020).

The current study has several limitations, which necessitate highlighting areas for future research. First, our study was conducted on a 14 m indoor runway and did not examine changes in gait parameters during prolonged running. While this protocol is clinically validated, its short duration inherently minimizes the accumulation of integral drift, a known limitation of IMU-based spatial analysis. Consequently, the high accuracy observed in this study must be interpreted within the context of short laboratory distances where drift accumulation is minimal. Future studies should incorporate long-distance running experiments to more convincingly validate the accuracy of gait parameters to ensure their applicability in real-life running gait analysis (Straczkiewicz et al., 2023). Second, the current validation focused on a specific cohort of healthy young males. It should be noted that the validity of the algorithmic parameters within the MFD-GED framework is currently established strictly within the context of the gait characteristics of this specific cohort. Since biomechanical variables (e.g., impact loading and limb swing velocity) vary significantly with gender and age, the direct application of the current framework to other populations might affect detection performance. Therefore, future deployment in broader cohorts (including females and older adults) necessitates verifying the suitability of these parameters, potentially requiring population-specific tuning to ensure optimal accuracy. Additionally, future studies should assess the algorithm’s robustness in individuals with gait pathologies (e.g., asymmetry or deformity) to verify the generalizability of the parametric event detection strategy. The next applications involve adapting this algorithm for real-time running gait analysis in sports settings. Furthermore, a significant methodological limitation was the absence of hardware synchronization (e.g., TTL triggering) between the IMU sensors and the laboratory reference system. Consequently, a direct, point-by-point validation of the absolute timestamps for individual IC and TC events could not be performed. However, it is important to note that this limitation does not compromise the validity of the derived gait parameters, as these metrics rely on relative time intervals rather than absolute time registration. Future improvements to this approach could include better balancing between angular velocity and acceleration data for more precise event detection and gait parameter estimation. Ultimately, we aim to develop a real-time foot-mounted IMU gait monitoring and analysis system applicable to real-world scenarios, contributing to advancements in running gait analysis technology.

## 5 Conclusion

The present study introduces a novel method for accurately estimating gait spatiotemporal and kinetic parameters in running gait analysis via a single foot-mounted IMU. A fusion-based and parametric approach, MFD-GED, was used to increase event detection accuracy. The method underwent comprehensive validation, including Pearson’s r, ICCs, and Bland–Altman analysis, providing a more systematic evaluation than commonly seen in previous IMU-based method development. The findings from the 10-m run test confirm the high validity of the MFD-GED method, demonstrating its effectiveness for detailed running gait characterization within controlled environments. Compared with those of previous methods, the accuracy of the estimation is improved by selecting the optimal gait segmentation and event detection algorithms. Although the estimation accuracy for FT and peak vGRF was slightly lower than for other metrics, the overall performance indicates that the MFD-GED method is sufficiently robust for biomechanical analysis in research applications. Notably, while currently validated in an offline mode for healthy young males, our next steps involve extending this framework to more diverse populations and running conditions. Ultimately, our aim is to develop a portable gait monitoring and analysis system applicable in real-world scenarios, contributing to advancements in running gait analysis technology.

## Data Availability

The original contributions presented in the study are included in the article/supplementary material, further inquiries can be directed to the corresponding author.
